# The Clinical Relevance of Interdisciplinary Research on Affect Regulation in the Analytic Relationship

**DOI:** 10.3389/fpsyg.2021.718490

**Published:** 2021-10-15

**Authors:** Carolina Altimir, Juan Pablo Jiménez

**Affiliations:** ^1^Faculty of Psychology, Universidad Alberto Hurtado, Santiago, Chile; ^2^Millennium Institute for Research in Depression and Personality (MIDAP), Santiago, Chile; ^3^Psychiatry Department, Universidad de Chile, Santiago, Chile

**Keywords:** epistemology, psychoanalytic research, therapeutic relationship, affect regulation, interdisciplinarity, clinical relevance

## Abstract

After more than a century of existence, theoretical development, research, and clinical practice within the psychoanalytic movement have consistently demonstrated that psychoanalysis is not a unitary and autonomous discipline. This has been evidenced by the various ways in which psychoanalytic thought and practice have been informed by and have established a dialogue—more or less fruitful—with related disciplines (neurosciences, developmental psychology, psychotherapy research, attachment theory and research, feminism, philosophy). This dialogue has contributed to a better understanding of the functioning of the human psyche, and therefore of the analytic process, informing clinical interventions. In turn, it has enriched research on psychoanalytic practice and process, underlining the fact that research in psychoanalysis is fundamentally about clinical practice. Since its origins, psychoanalysis has made explicit the work on the patient-analyst relationship as the terrain in which the analytic process unfolds. For its part, research in psychotherapy has demonstrated the relevance of the therapeutic relationship for the good development and outcome of any psychotherapeutic process. This supports the argument that research in clinical psychoanalysis should be research on the impact of the analyst interventions on the analyst-patient relationship. In this context, a central element of what happens in the analytic relationship refers to affect communication and therefore, affect regulation, which is manifested in the transferential and counter-transferential processes, as well as in the therapeutic bond. On the other hand, affective regulation is found at the crossroads of etiopathogenesis, complex personality models and psychopathology, allowing the understanding of human functioning and the staging of these configurations in the patient-analyst relationship. In this way, research on affective regulation in the analytic process is proposed as a path that exemplifies interdisciplinary research and scientific pluralism from which psychoanalysis enriches and progresses as a discipline. The case of a line of research on affective regulation in psychoanalytic psychotherapy is illustrated. The need to resort to other disciplines, as well as the translational value of our research and its clinical usefulness, is discussed.

## Introduction: Psychoanalysis Is Neither an Autonomous nor a Unified Discipline

In this paper we propose a shift in psychoanalytic research from meta-theory to focus on the immediacy of the psychoanalytic encounter. In this, we follow the scientific program proposed by Joe Sandler (1983) to investigate “what analysts do” in their clinical practice. We formulate this program more precisely by stating that our research should seek to capture “the practice of psychoanalysts on its own merits” (Jiménez, 2009). However, this goal requires the application of methodologies that go beyond the traditional clinical method and that integrate clinical/hermeneutic exploration, systematic empirical research, and interdisciplinary findings from other mind/brain disciplines. Our line of research intends, precisely, to present a novel methodology that allows us to approach the immediacy of the encounter between analyst and patient. By the way, our method does not pretend to be the only possible method, but we hope that throughout this paper its advantages will become clear.

The paper begins by describing the shortcomings of psychoanalysis as a scientific discipline, as a result of its secular isolation from other disciplines of the mind. Then, even if it seems contradictory, we argue that since Freud’s time, the findings of the other disciplines of the mind have inadvertently “infiltrated” the theoretical construction of psychoanalysis. Since its very inception, interdisciplinary findings have played a role in psychoanalytic knowledge. Indeed, a fundamental contemporary influence on psychoanalytic thinking is the shift toward the relational perspective. However, the challenge for research is to develop methodologies that can capture the relational and intersubjective character of the analytic dyad. In the following we suggest that the study of affect self-and dyadic regulation within the therapeutic relationship is a window to study emergent phenomena in the interactive “in-between” of the interaction. Finally, we present our approach by illustrating some pieces of research where patient and therapist facial-affective behavior and its regulatory function within ruptures and repairs of the relationship are examined. We propose this observational approach to study the patient-analyst interaction as one possible pathway that may contribute to the systematic research on analytic process.

The epistemological status of the psychoanalytic discipline, as a natural science or as a social science, as both or in between, is still a matter of controversy after more than 100 years. Freud always considered the possibility of a unified psychoanalytic science; for decades, the building of a psychoanalytic theory was dominated by the assumption that the accumulation of clinical knowledge, the third pillar of the Freudian definition of psychoanalysis, would lead to the construction of a unified scientific discipline (Freud, 1923). From a current perspective, however, the possibility of that becoming a reality was never real; from its birth, psychoanalysis evidenced divergent theoretical and practical points of view (Makari, 2008).

In the last decades, a consensus has been reached that psychoanalysis is not a unified clinical or theoretical discipline. We argue that one of the main reasons for this is the use of the psychoanalytic clinical method as the sole source of psychoanalytic knowledge (Jiménez, 2015). This has favored a tendency toward fragmentation and the development of multiple and ramified theories that do not converse among one another. Accordingly, practical and theoretical diversity constitutes an inevitable fact in psychoanalysis. For a century this diversity was constrained by referring to authority as a means to place the inherent tendency to diversification into a straightjacket. However, sharp questions emerge: What can we do in the face of the growing plurality of orientations and positions in psychoanalysis? Is psychoanalytic knowledge doomed to an endless fragmentation? In more than 100 years, experts have been unable to agree on how to define core concepts such as “psychoanalytic process” or even the very concept of “psychoanalysis.” What are the tasks involved in the construction of psychoanalytic pluralism? Is psychoanalysis doomed to disappear as a discipline, i.e., as a unified “branch of knowledge, typically one studied in higher education,” as The Oxford English Dictionary defines “discipline”? In the university tradition an academic discipline supposes some central elements like the presence of a community of scholars; a tradition or history of inquiry; a mode of inquiry that defines how data is collected and interpreted, as well as a definition of the requirements for what constitutes new knowledge; and the existence of a communication network. But, to what extent does contemporary psychoanalysis meet these requirements? Precisely, the contemporary controversy in psychoanalysis centers around the mode of inquiry, hermeneutic or scientific, or both, that defines how data is collected and interpreted, and the requirements for what constitutes new knowledge in psychoanalysis; in short, the validity of knowledge that is based solely on clinical method. All these are questions that continue without definitive answers.

Over the past 20 years, however, a growing consensus about the nature of the epistemological and professional crisis in psychoanalysis has emerged. Even though in the past two decades many experts (see Kandel, 1998, 1999; Kernberg, 2012; Thomä, 2015; Kernberg and Michels, 2016; Schachter and Kächele, 2017) have advocated for the insertion of psychoanalysis within universities, to bring innovation into psychoanalytic education, to broaden the conception of psychoanalysis in order to include the diversity of psychoanalytic psychotherapies, to establish theoretical bridges with cognitive psychology and the neurosciences, to widen the basis of the theory beyond the clinical method toward the social and natural sciences research methods, to name but a few; the most psychoanalysts, who work in the isolation of their private practice, have not been persuaded.

Faced with this situation of paradigmatic crisis, Joe Sandler proposed that “psychoanalysis is what psychoanalysts practice” (Sandler, 1982, p. 44). This statement seems tautological, because certainly, not everything a psychoanalyst practices is, by that fact alone, psychoanalysis. We believe that this statement is rather a research program of clinical practice, a call to explore “the practice of psychoanalysts on its own merits” (Jiménez, 2009), that is, in a valid way. This responds to what empirical research in psychotherapy and psychoanalysis has evidenced: that what psychoanalysts do is different from what they say they do. Nevertheless, the latter is what is discussed and exchanged in clinical and scientific meetings. The gap that is thus constituted, between actual practice and an idealized psychoanalytic practice, is what prevents the controversy about what psychoanalysis is from ever coming to consensual terms.

## Psychoanalytic Hermeneutics and Psychoanalytic Science: The Role of Interdisciplinary Knowledge

It seems that within psychoanalysis, a dichotomy between hermeneutics and science still strongly prevails. This is partly due to the fact that scientific inquiry is assumed to be reduced to a pure positivist, quasi-experimental empiricism. This notion of science and the consequent criticism to its value for psychoanalysis, is in part consequence of the proliferation of randomized clinical trials (RCT) as a method for testing the effectiveness of psychotherapies, as well as the underlying evidence-based practice model promoted by healthcare providers and funders (Safran, 2012). Inasmuch as some of the criticisms toward the limitations of this approach to psychotherapy research can be shared, the rejection toward this particular method has penetrated the entire notion of systematic empirical research. Thus, to talk about psychotherapy research is equivalent to “desiccate human experience,” as RCT models would not recognize the uniqueness of every analytic dyad (Safran, 2012). Indeed, claims from the same psychoanalytic community that advocate for the incorporation of rigorous and systematic research procedures, in addition to the clinical case study method, for the generation of psychoanalytic knowledge (Wallerstein, 1993), have been criticized as reductionist, empiricist, and positivist. As McWilliams (2011) argues, the “self-defeating political legacy of many analysts’ contempt for research on the analytic process” (p. 9) remains despite empirical work that has shown the effectiveness of analytic treatments. According to McWilliams, “Many scholars prefer to place psychoanalysis within the hermeneutic rather than the scientific tradition, partly because of this resistance of much of the subject matter to investigation by the scientific method as it has come to be defined by many contemporary academic psychologists” (McWilliams, 2011, p. 23).

The criticism of the legitimacy of empirical process and outcome research is shared by both classical and postmodern psychoanalysts (Jimenez and Altimir, 2019). In fact, several postmodern authors have argued that systematic empirical research has little to contribute to the practice of psychoanalysis (Hoffman, 2009, 2012; Stern, 2013), and that it can poorly capture what takes place in the intersubjective encounter of the analytic situation (Orange et al., 1997), thus rejecting extra-clinical research as a legitimate source of psychoanalytic knowledge.

Several important researchers who are also psychoanalysts have made efforts to broaden the perspective and definition of what constitutes systematic research in psychotherapy and psychoanalysis, advocating for a constructive dialogue between hermeneutics and scientific research (Safran, 2012; Fonagy, 2013; Strenger, 2013), and thus avoiding what Safran (2012) has called the “tendency toward insularity” that dominates psychoanalysis. These authors have described several forms of research, alternative to the RCT, to describe and understand psychotherapy and psychotherapy process. These include qualitative research, research on the mechanisms of change, the study of specific therapy events that involve change or are associated to relevant aspects of therapy, and systematic and rigorous approaches to single case studies (Fonagy and Moran, 1993; Messer, 2007; Szecsödy, 2008; Safran, 2012). This broader understanding of what the scientific endeavor involves, includes not only confirmation or refutation of assumptions regarding psychotherapy process and outcome, but also, and perhaps most importantly, the process of meaning-making in the interpretation of research findings (Fonagy, 2013). As Safran (2012) suggests, this can contribute to a broader understanding of the way in which science actually works, which is nurtured by a dialogue among members of a scientific community.

Along with many, we have come to the conviction that, in order to develop as an academic discipline and as a recognized and legitimated profession, psychoanalysis must cultivate not only its hermeneutic aspect, but also its scientific side. This means a simultaneous emphasis on examining analytic process and outcome, as well as the incorporation of the interdisciplinary study of the mind/brain relationship, and its interplay with development, personality, and psychopathology in the understanding of our patients. It is our contention that only the interdisciplinary scientific study of psychoanalytic insights may contain within limits the tendency to fragmentation inherent in the interpretive psychoanalytic method. Here, we agree with Carlo Strenger (1991) when he argues that theoretical propositions must not only be coherent, but they should also be consistent with a body of knowledge that is generally accepted and incorporated to related disciplines.

As stated in the introduction, practical and theoretical diversity constitutes an inevitable fact in psychoanalysis since is a complex field, where there is no room for linear or simple understandings. Thus, one of the basic assumptions of complex phenomena is that their study should be interdisciplinary in nature (Kendler, 2005; Morin, 2008). In two books that had a strong impact at the time, Henri Ellenberger (1970) and Frank Sulloway (1979) demonstrated that beyond the Freudian legend, since Freud interdisciplinary dialogue has never been foreign to the formation of theory in psychoanalysis. In contemporary times, and as we showed in a recent review dedicated to the subject (Jimenez and Altimir, 2019), this has been evidenced by the various ways in which psychoanalytic thought and practice have been informed by and have established a dialogue—more or less fruitful—with related disciplines (neurosciences, developmental psychology, psychotherapy research, attachment theory and research, feminism, philosophy). All these disciplines have emphasized the interactive and relational character of human development. Increasingly, psychoanalytic theory and practice have incorporated the findings of related disciplines into their conceptualization of human development and psychopathology, of the mind/brain relationship and of the relational processes involved in psychotherapy. The “contamination” of clinical knowledge with findings from related disciplines of the mind has been a slow and surreptitious, but a very effective process. Thus, Canestri notes that the field is coming to “a redefinition of the object of (psychoanalytic) study; that is, the particular intersubjective figure constituted by the analyst-patient relationship” (Canestri, 1994, p. 1079). Nevertheless, empirical research in psychoanalytic therapy has not done justice to the “dyadic nature of the construction of experience” during therapy. Although the idea of defining empirical variables based on relational concepts may seem obvious, in practice a great amount of research efforts interested in the therapeutic relationship do not fully account for the relational essence of this phenomenon.

## The Relational and Intersubjective Character of the Analytic Dyad

Since the 1980s, the field of psychotherapy has experienced a major shift toward a relational perspective (Aron, 1996; Muran and Samstag, 2008), from which psychoanalysis has not been exempt. Amidst the theoretical and technical diversity of contemporary psychoanalysis, a prolific discussion has developed around the intersubjective nature of analytic work. Several theoretical orientations within the umbrella of contemporary psychoanalysis have developed or emphasized concepts that account for the interest in what happens in the interaction between patient and analyst (Foehl, 2010; Bohleber, 2013). Thus, Lewis Aron (1996) refers to “relational psychoanalysis” or “relationally oriented therapies” to refer to the group of theories within psychoanalysis whose main focus of interest is relationships, emphasizing both intrapersonal and interpersonal relationships.

Within the wave of the relational movement, contemporary psychoanalysis has been influenced by the postmodern turn toward constructivist and intersubjective thinking (Aron, 1996; Bohleber, 2013). From the postmodern viewpoint, the world is uncertain, so that no general principles about human nature can be established. Thus, “reality” or “truth” is not one and unique but depends on who experiences/observes it (Wachtel and Messer, 1997). From this perspective, the object of experience is never separate from the subject who experiences it. Thus, the aspiration to separate the knowing subject from the knowing object is replaced by the idea of a subject-subject relationship, in which intersubjective reciprocity is inevitable (Foehl, 2010; Bohleber, 2013). Gadamer’s (1966) hermeneutic perspective in turn has been incorporated by relational thinking by emphasizing that the subject’s perception of reality is always influenced and thus constrained by his/her preconceived ideas and prejudices (hermeneutic circle). As Foehl (2010) argues, the implication of this perspective is that we humans experience one another from a position that shifts and changes as we engage.

From this point of view, the Freudian aspiration to eliminate the subjective factor from the analytic process is questioned and reformulated, toward the idea of being able to study and recognize the subjective factor *within* the analytic encounter (Aron, 1996). The questioning of clinical neutrality and abstinence has also been significantly influenced by the empirical findings from attachment, developmental, and neuroscientific research over the past five decades. These fields have gathered substantial evidence indicating the interactive nature of the development of the mind and brain (Allen, 2013; Schore, 2013). Furthermore, developmental neuroscience supports the notion that the infant brain is designed to be shaped by the social environment in which it develops (Thomas et al., 1997), and in that sense, it is considered to be a “social brain” (Brothers, 1990). The main implication derived from this conception of human development is that just as infant and caregiver co-construct their subjective experience of the world, of self and other, patient and therapist also co-construct their experience of the therapeutic relationship and of the emotional exchange involved in transference and countertransference processes (Aron, 1996; Schore, 2016). Thus, the patient-therapist relationship is the object of study, and the therapist is considered a co-participant, rather than someone who can stand outside the interpersonal field and observe from there (Aron, 1996).

At the same time, decades of research in affective neuroscience have questioned the supremacy of cognition in human information processing and development, and have emphasized the importance of affect, implicit memory and procedural phenomena in mental functioning (Damasio, 2005; Panksepp, 2005). This implies a recognition and acceptance of subjectivity in neurobiological research (Schuessler, 2003). Kendler (2005) proposes that subjective or “first-person” experiences have causal efficacy in the body and can be understood as highly elaborate forms of intentional processes that eventually lead to action and result in achievements such as language, customs, technology, and culture. Mental disorders emerge from the failure of these intentional states to exert effective action in the world (Spence, 1996). In this regard, Fonagy (2003, p. 108; italics in original) argues that “*Intrapsychic representational processes are not just consequences of environmental and genetic effects*—*they may be critical moderators*. […] The primary evolutionary function of attachment may be the contribution it makes to the creation in the individual of a mental mechanism that could serve to moderate psychosocial experiences relevant to gene expression.” In other words, he states that the *interpretation* of the social environment and not the mere “objective,” physical environment acts on genetic expression. The subjective perception of the social environment (e.g., perception of isolation or social anxiety) can generate changes in several levels of the body’s response systems, such as the central nervous system, hypothalamic pituitary adrenal axis, intracellular signals, and finally transcription factors and genetic expression (Slavich and Cole, 2013).

Considering the abovementioned developments, the analytic process and the patient-analyst relationship becomes the focus of clinical interest and, following our proposal, of research inquiry. Given that there is no consensual definition of what a psychoanalytic process is essentially, and both the traditional inquiry based on the clinical case, as well as empirical approaches have reached a stalemate (Altimir and Jiménez, 2020), in our proposal we adopt an observational stance. We understand the analytic process as that which transpires in the interaction between patient and analyst throughout time. With this respect, we agree with Schachter and Kächele’s (2017) conclusion that since it is not possible to define or measure the traditional concept of psychoanalytic process, research must change strategies and focus instead on the detailed observation and description of this interaction. Based on this perspective, we ask ourselves which interactions and the mechanisms involved in them relate to patient change and which do not. This approach also takes into consideration, precisely, the perspective underscored by the relational turn, where this interaction cannot be understood without the influence of the person of the analyst.

Foehl (2010) argues that this perspective has meant a shift from meta-theory to a focus on the immediacy of the analytic encounter, suggesting that it is time for psychoanalytic inquiry—and we would also add systematic investigation of the analytic process—to move from a prescription of what the content of the analytic process should be, toward a focus on describing the structure of the process from a stance close to experience. This means looking at the performative dimension of clinical practice. In the same vein, Schachter and Kächele (2017), in a critical review of psychoanalytic training and theorizing, conclude that it is not possible to define and measure the concept of psychoanalytic process from a top-down (i.e., prescriptive) perspective, but that it is necessary to shift the strategy toward a focus on detailed and systematic observation and description of the patient-therapist interaction. This includes the ways in which subtle or implicit (unconscious) interactions and enactments may dominate the clinical situation, and how the subjective experiences of patient and therapist are influenced by the implicit actions and gestures of the other (Aron, 1996). This includes the affective processes involved in the development, maintenance, and regulation of the therapeutic relationship (Benecke et al., 2005).

## Psychotherapy Research Supports the Relevance of the Analytic Relationship

Following Strenger’s (1991) argument, we consider that any conceptual proposition as well as systematic inquiry of the analytic process and the patient-therapist relationship must be consistent with the cumulative body of knowledge generated by nearly 50 years of psychotherapy research. After decades of the so called “legitimacy studies,” the field has come to the conclusion that psychotherapy is effective, and that, on average, there are no significant differences in effectiveness between different types of psychotherapy. This has been called the “paradox of equivalence,” leading to further studies that attempted to identify the different therapy factors that explain outcome and client change (Wampold and Imel, 2015). The findings from the numerous effectiveness studies have indicated that there is a significant proportion of the variance explaining therapy outcomes that is due to common or non-specific factors, that is, to elements that are involved in the therapist’s contributions to the treatment and above all, relationship factors (Wampold, 2001; Lambert, 2013). Lambert (2013) estimated that the therapeutic relationship and patient expectations (both non-specific placebo effects) were responsible for 45% of that of improvement.

In the attempt to specify what are the active ingredients that make up the realm of the unspecific therapy factors, psychotherapy research has since examined the elements of the therapeutic relationship that contribute to change and outcome. After four decades of prolific research, findings indicate that the most important generic factor of change is the therapeutic alliance (Wampold and Imel, 2015). What is perhaps the most relevant aspect of these findings is the consistency of the alliance-outcome relationship (i.e., alliance as a robust predictor of outcome) across treatment orientations, patient disorders, rating methods for both alliance and outcome (i.e., client and therapist self-reports and observational measures), and across contexts in which the treatment is delivered (i.e., naturalistic settings as well as manualized treatments) (Martin et al., 2000; Horvath and Bedi, 2002; Horvath et al., 2011; Flückiger et al., 2018; Del Re et al., 2021). This can only confirm that the elements that compose the alliance, as it has been measured throughout these studies, constitute a significant portion of what transpires in the therapeutic exchange. These elements include the mutual agreement between patient and therapist on the tasks and goals of treatment, as well as an affective bond between them (Bordin, 1979), generating a collaborative relationship. Thus, the relevance of the therapeutic relationship for therapy process, and specifically for the analytic process that is our focus of interest, becomes unquestionable. This comes to confirm, through extra-clinical systematic research, the primacy psychoanalysis has given since its birth to the patient-analyst relationship as the via regia for the psychoanalytic process to unfold.

In an attempt to address the question of what specific elements are involved in this relational process, prominent exponents of the so-called “second generation” of alliance research, have redefined the alliance, suggesting “an inextricable relationship between the technical and the relational—that every intervention has relational meaning. It also suggested a more mutual and dynamic process of ongoing negotiation, which stands in contrast to previous conceptualizations that emphasized the therapist’s support or the patient’s identification with the therapist and acceptance of the therapist’s values for the psychotherapy process” (Muran et al., 2010, p. 321). These researchers and clinicians have been interested in studying rupture-repair processes within this ongoing negotiation, based on which they have developed a therapeutic model focused on the alliance (Muran et al., 2021). Thus, they have managed to combine research with clinical practice, facilitating interdisciplinary dialogue. They not only base their clinical principles on relational thinking, but they have also been informed by mother-infant research on affect regulation and interpersonal complementarity. Although they adopt Bordin’s definition of the alliance, Safran and Muran (2006) have questioned the use of the concept in the terms in which it has been studied, as it would over-emphasize the role of conscious collaboration, and in turn underestimate the pervasive role of unconscious factors in patient and therapist co-participation in the therapeutic exchange. They argue that there is no longer a need to use the concept of alliance to distinguish it from transferential and counter-transferential components of the relationship, as it was originally regarded by Freud, since any attempt to disentangle the technical from the relational dimensions of therapy would be conceptually problematic (Safran and Muran, 2006). Instead, they propose the notion of negotiation to suggest “that the alliance concept can include a view of the psychotherapy process as involving an ongoing push and pull of various patient and therapist affective states, underlying needs, and interpersonal behaviors” (Muran et al., 2010, p. 321). Along this dialectic play between the patient’s and the therapist’s positions, accommodations, and hostilities, as well as contradicting needs of both participants are being exchanged. This conveys the fact that the world consists of others with separate subjectivities and that these subjectivities are potentially negotiable, without the need to deny the other’s or one’s own. In this regard psychotherapy involves an intersubjective negotiation in which both participants are engaged in a struggle for mutual recognition of their respective subjectivities (Muran et al., 2010). During this process affective states play a central role (Muran and Samstag, 2008), as they have been observed to be highly relevant in establishing and shaping the therapeutic relationship, serving a regulatory function (Benecke et al., 2005).

## The Role of Affect Regulation in the Analytic Relationship

We propose that affect regulation between patient and therapist constitutes a phenomenon of particular interest for the research of the analytic process, since it is inextricably involved in the intersubjective negotiation of the therapeutic relationship. In the relational psychoanalytic literature, the notion of intersubjective negotiation contains the idea that patient and therapist mutually negotiate affective states (Muran and Samstag, 2008). Affective processes are inherent to the exchanges that constitute and configurate the therapeutic relationship (Benecke et al., 2005). Thus, we assume that in the analytic exchange, affect regulation involves a process through which both patient and analyst experience and regulate internal affective states, while they express them through verbal and non-verbal channels. In doing so, they adopt a communicative value within that specific relational context, serving as information about the interactive partner’s experience and therefore influencing the concomitant affective response. These responses maybe more or less accurate in apprehending the partner’s subjective experience, and thus the relationship can oscillate across different levels of affective coordination and subsequent reparation of miscoordinations (Tronick, 2007; Fonagy, 2015). Nevertheless, affect regulation has been scarcely targeted as a process susceptible of being systematically examined from a research perspective as it unfolds in the analytic process.

We believe this responds to a characteristic that makes affect regulation a challenge as well as an opportunity for interdisciplinarity in psychoanalytic research. Affect regulation is situated at the crossroads of cognitive sciences (Gross, 2014), neuroscience (Schuessler, 2003; Schore, 2012; Gyurak and Etkin, 2014), developmental psychology and attachment (Bowlby, 1969; Beebe and Lachmann, 2002; Fonagy et al., 2002; Tronick, 2007), etiopathogenesis and personality (Blatt, 2008; Blatt and Luyten, 2009), psychopathology, psychiatry, and psychotherapy (Fonagy et al., 2002; Schore, 2012). The interest of these variety of disciplines in affect regulation responds to the increasing acknowledgment of its central relevance for the operation of the human mind and its relationship to the environment (Fonagy et al., 2002; Schore, 2012; Gross, 2014; Taipale, 2016), and to the development of adaptive as well as maladaptive mental functioning (Berenbaum et al., 2003; Berking et al., 2019). At the same time, this has implied a conceptual diversity that challenges the possibility of a unitary or simple definition.

Nevertheless, this diversity may be an opportunity of interdisciplinary dialogue as well. Here, we propose that the attempt to examine and understand the analytic relationship from the perspective of affect regulation, opens a field of inquiry that can contribute to deepening the understanding of how implicit and unconscious mental states can acquire complex meaning by means of an intersubjective exchange. To the extent that contemporary psychoanalysis has been emphasizing the importance of focusing on the immediacy of the experience of and with the patient (experiential turn), it has become more important to pay attention to the emotions that flow in and in between patient and analyst. This inevitably has the technical consequence of accentuating an observational attitude on the part of the therapist, before giving way to interpretative actions (phenomenological turn). It is precisely this tendency that makes self- and dyadic affect regulation a particularly important point in contemporary psychoanalysis (Jiménez, 2007). This is in agreement with interdisciplinary findings. In line with this, from our perspective, affect regulation is relevant both form a clinical as well as an investigative stance, to understand analytic process and the therapeutic relationship by way of different connecting pathways.

First, as developmental psychology and attachment research have indicated, affect regulation is an essential element in the process of the development of the self during early infancy. Thus, affect regulation contributes to clinical formulation by providing a comprehensive paradigm based on the “observed infant” that informs clinical work with the “clinical infant” (Stern, 1985). Several models within the developmental and attachment fields of research indicate that affect regulation constitutes a central process involved in the innate motivational attachment system (Beebe and Lachmann, 2002; Fonagy et al., 2002; Schore, 2012; Allen, 2013; Taipale, 2016). The infant-caregiver relationship is characterized by a highly efficient and essentially non-verbal system of emotional communication through which affect is transacted, whose function is to regulate the changing levels of the infant’s arousal and emotional states (Beebe et al., 2005; Allen, 2013; Schore, 2016). Early affect regulation is carried out by the primary caregiver, who reads the baby’s automatic emotional expressions and reacts with an affective mirroring, that allows the child to correctly attribute these states to him/herself and distinguish them from the caregiver’s, fostering the capacity for affect self-regulation (Gergely and Watson, 1999; Watson, 2001; Gergely, 2007). The quality of how affect is reflected impacts the development of the processes of emotional regulation and self-control, including mechanisms of attention and voluntary control. Along these progressive exchanges between infant and caregiver, the infant moves from a state of co-regulation, depending on the caregiver’s ability to contain and mirror the child’s affective states, to self-regulation, and therefore the regulation of the interactions with others (Fonagy et al., 2002; Beebe et al., 2005; Taipale, 2016). This continuous process enables the infant to develop a second order system of representation for mental states (Fonagy and Target, 2002). This will largely determine the child’s ability to develop representations of self and others as separate entities with different intentions, desires, and feelings (Gergely and Watson, 1999; Watson, 2001; Fonagy et al., 2002; Gergely, 2007), This intersubjective process results in a gradual organization of emerging self-states in configurations of actions and responses that will result in the individual’s sense of self and others (Allen, 2013). This process is the basis for intersubjectivity and therefore for the capacity for mentalizing (Fonagy et al., 2002; Bateman and Fonagy, 2006). Mentalization is defined as the achieved ability to conceive of oneself and others as possessing beliefs, feelings, attitudes, desires and intentions and therefore to give meaning and predictability to the behavior of others (Allen et al., 2008). Thus, the child’s ability to develop representations of themselves and others as separate entities with different intentions, desires and feelings will translate into particular configurations of psychological functioning throughout the lifespan (Fonagy et al., 2002; Mikulincer and Shaver, 2007), which will include specific modalities of self and mutual affect regulation. This supports the notion that in infancy and along the human lifespan, the regulation of affect is a central organizing principle of human development and motivation (Schore, 2003).

Second, the result of the developmental pathways to adulthood manifest in relatively stable representations of self and others, are considered by several authors as the foundations of the development and functioning of the personality (Meyer and Pilkonis, 2005; Pietromonaco et al., 2006; Luyten and Blatt, 2015). Specifically, they constitute cognitive-affective interpersonal schemas of self and others, that can range from relatively broad representations applicable to various situations, to more relationship-specific representations (Luyten and Blatt, 2011). In turn, they constitute the building blocks of the individual’s capacity to establish and maintain reciprocal, meaningful, and personally satisfying interpersonal relationships with others; and to establish a coherent, realistic, differentiated, and essentially positive sense of identity (Luyten and Blatt, 2011), that it, between interpersonal relatedness and self-definition (Allen, 2013).

The aforementioned concepts have in turn permeated the development of complex dimensional models of personality and etiopathogenesis, that have emphasized the importance of contemplating developmental processes, and of integrating findings from epigenetics and developmental psychology (Jiménez et al., 2018), in response to the limitations posed by categorical and disorder-centered proposals (Widiger and Samuel, 2005; Krueger and Markon, 2006; Clark, 2007; Blatt and Luyten, 2010). From this perspective, emphasis is placed on the consideration of the various developmental pathways of psychopathology that include genetic, temperamental and personality dimensions, and their interaction with the environment, in the shaping and consolidation of altered cognitive-affective schemas of the self and others throughout the lifespan (Blatt and Luyten, 2009, 2010). This approach implies a fundamental change of perspective, by considering an understanding based on the expression of subjectivity and not only of symptomatology, and which understands this expression as a complex psychological process resulting from a specific trajectory (Allen, 2013; Luyten and Fonagy, 2019).

These complex dimensional models of personality propose, in different ways, that the relatedness and self-definition dimensions are the key psychological coordinates of human functioning as well as of normal and disrupted personality development (Luyten and Blatt, 2011, 2013; Skodol et al., 2011). Furthermore, following Allen (2013), the dialectic among these dimensions intimately relates with the interactive processes involved in early attachment relationships and that consolidate differing levels of the development and regulation of the self. Specifically, the adult personality configuration will be characterized by certain affective regulatory strategies put forward in the context of close relationships (Meyer and Pilkonis, 2005; Pietromonaco et al., 2006; Mikulincer and Shaver, 2007).

Third, inasmuch as affects and their regulation constitute a fundamental process for the development of the self and of the emotional interactive repertoires employed to negotiate relationships throughout the lifespan, the therapeutic relationship will constitute a new scenario where these processes will necessarily unfold. The negotiation of the needs for agency and relatedness will be manifested through specific affective regulatory strategies, which may include self-regulation as well as hetero-regulation, that is, an invitation for the interactive partner to help regulate emotional arousal and dysregulation. Again, this will be shaped by the distinctive configurations of each participant’s sense of self and others and the levels of anxiety involved in the interpersonal connection. In the face of particular patient-therapist transactions that generate some level of dysregulation on the patient, predominant attachment patterns (and therefore attempts to regulate the concomitant affective states) will be experienced and reenacted in the therapeutic relationship (Diamond et al., 2003; Allen, 2013), constituting an important component for the transferential and counter-transferential process. At the same time, these patterns will influence both patient’s and therapist’s capacities to tolerate frustration and anxiety in the analytic relationship as well as to explore ruptures of the alliance (Eames and Roth, 2000; Allen, 2013). In fact, evidence indicates that therapists’ capacity to regulate their own affect during ruptures of the alliance is crucial in the ability to address and repair alliance ruptures (Muran et al., 2010; Allen, 2013; Eubanks et al., 2018). These affect regulatory capacities are in turn intrinsically related to the process of mentalizing in the analytic relationship. According to Allen (2013), psychotherapeutic process, by means of the patient therapist relationship, favors improved mentalizing, a capacity that emerges from the repeated process of understanding and being understood. In his view, psychotherapists “are mentalizing and engaging their patients in the process of mentalizing while endeavoring to provide their patients with a safe relationship that bears the hallmarks of secure attachment. Moreover, to the extent that the therapy addresses problems in close relationships—including ways of relating to oneself—the content of the therapy process pertains to attachment” (p. 163). Inasmuch as mentalizing involves having the other’s mental states in mind, it contributes to the process of mutual affect regulation within the analytic dyad.

In this section we have argued about the centrality of affective (self-) regulation in intimate relationships, the paradigm of which is the early mother-infant relationship, bringing together arguments from different disciplines of the mind. One of the basic intuitions of psychoanalysis has been that the analytic relationship reproduces, in some way, the early relationship that the patient had with her/his mother (transferential patterns). However, Daniel Stern emphasized that the representation of the “clinical baby” on which the analyst focuses his therapeutic work should not be confused with the “observed baby,” there being crucial differences between the two configurations. In the same vein, empirical research, which has been prodigal in comparisons between the clinical baby and the observed baby, has not achieved the same development in the investigation of the therapeutic relationship “on its own merits” (Jiménez, 2009). Our proposal, therefore, suggests that the focus of the empirical study of the psychoanalytic relationship should be on the mutual (self-) regulation between patient and analyst, which constitutes a privileged window for its study.

## How to Investigate Affect Regulation in the Analytic Dyad: An Illustration

In the attempt to make a case for the contribution of interdisciplinary research on affect regulation in the analytical relationship, we will describe a line of research developed by the first author. Based on the arguments posed above, this line of research is interested in studying mutual and self-affect regulation within the therapeutic dyad during particular relational events that deem relevant for the negotiation of the relationship and therefore for the therapeutic process. Therefore, we have drawn on the Rupture Resolution Model developed by Safran and Muran (2000), which comprises a combination between psychotherapeutic process research on ruptures and resolutions as well as a relationally sound and empirically informed model for therapy centered on working on the therapeutic relationship. We have already described the relational background that also informs this model. Nevertheless, we want to stress that the rationale behind the selection of these relational events within therapy responds to the fact that they are relevant and discrete instances or “windows” to the intersubjective negotiation process in its fullest manifestation.

Alliance ruptures have been defined as temporary deterioration in the alliance manifested by a disagreement between patient and therapist on the goals of therapy, lack of collaboration on therapy tasks or a strain in the emotional bond (Eubanks et al., 2018). At a simultaneous level, that have been defined as breakdowns in the continuous -conscious and unconscious- process of negotiation of patient and therapist’s respective needs, desires, and subjectivities (Muran and Eubanks, 2020). Therefore, ruptures involve the activation of dysfunctional relational patterns commanded by the participants’ relational schemes (Safran and Kraus, 2014). These relational schemes are manifest in the display of idiosyncratic affective repertoires, in an attempt to manage the emotional dysregulation caused by the relational impasse, expressed both verbally and non-verbally (Schore, 2011). During these relational events, it is possible to assume that each participant enacts their learned affective and relational repertoires founded on their representations of self and others, and of how relationships unfold (Safran and Muran, 2001; Beebe and Lachmann, 2002). These repertoires contain learned patterns that regulate affect and also determine expectations regarding the roles each member of the dyad must adopt in the interaction in order to respond to the specific needs for regulation (Bänninger-Huber and Widmer, 1999; Beebe and Lachmann, 2002).

According to the patient’s particular relational scheme activated, Safran and Muran (2000) have observed that ruptures can be expressed either as a withdrawal or emotional disengagement from the therapist, or the therapy process; or as a confrontation, where the patient expresses dissatisfaction in a non-collaborative way or attempts to control the therapist. It is relevant to note that ruptures are the results of both patient and therapist contributions, although their relative contribution may vary from case to case (Safran and Kraus, 2014). Therefore, they are co-constructed in this intersubjective process. At the same time, it is expected that each rupture, will adopt different forms depending on the particular characteristics of each participant in relation to their capacity to establish relationships (relatedness) and their capacity for self-definition (Safran and Kraus, 2014). This underscores the adequacy of the study of these events for the examination of the affect regulation process embedded in ruptures, as the participants’ strategies employed to address affective states within the therapeutic relationship will respond to these styles of functioning. In response to ruptures, the therapist may recognize and address them by implementing resolution strategies, which include direct (explicit acknowledgment) or indirect (implicitly resolving) attempts, as well as immediately focusing on the expeditious repair of the rupture in order to return to the original exchange, or expressive attempts that aim to shift the focus of the session to exploring the rupture and patient’s underlying needs or concerns (Eubanks et al., 2018).

These events are a prevalent phenomenon within therapy sessions (Eubanks et al., 2018; Muran et al., 2021), and can be considered interpersonal stressful events that challenge the stability of the relationship and the quality and progress of psychotherapy (Coutinho et al., 2011, 2014). Adequate management and positive resolution of ruptures is associated with greater benefits to patients, while a poor management of these events has been related to premature dropout (Tryon and Kane, 1995; Coutinho et al., 2011, 2014) and reiteration of ineffective interventions by the therapist (Castonguay et al., 1996).

The methodological challenge for the study of affect regulation within the analytic dyad is posed by the core elements that characterize affect and regulatory repertoires. As we have already reviewed in the previous sections, affect regulation is a process that initiates with birth and develops within the framework of the innate motivational system of attachment. The transactions involved in affect regulation are manifested through multiple channels of interaction (non-verbal, verbal, verbal, vocal, neuroendocrine, kinesthetic), resulting in multiple qualities of experience between baby and caregiver, and are organized into configurations of actions and regulatory responses (Tronick, 1989; Fonagy et al., 2002; Schore, 2005; Beebe, 2006). These transactions are initially organized in an implicit, procedural domain of experience, before the acquisition of language. This domain, sometimes called sub-symbolic (Bucci, 1997), encodes much of the procedural and emotional knowledge, which relates to the habitual way of establishing and negotiating interpersonal relationships. The sub-symbolic domain of experience is predominantly manifested through automatic non-verbal behavior. At the procedural level social behavior is regulated and coordinated moment-to-moment at the split-second level, largely outside of consciousness. The speed and density of information exchanged at this level does not allow for central control of cognition (Beebe and Lachmann, 2002). Most relational transactions rely heavily on a substrate of affective cues or signals that give an evaluative valence or direction to each relational communication. These communications are conducted at an implicit level of rapid signaling and response, occurring too quickly for simultaneous verbal translation and conscious reflection (Lyons-Ruth, 2000). However, the phenomena of transference and countertransference occur in response to these signals, and much of the pull and push of the relational negotiation will be manifest in this way (Schore, 2003).

Therefore, to access a relevant proportion of implicit affective states and their regulation, this line of research proposes to study patient and analyst’s facial affective behavior. Affects and affective states are mainly communicated through non-verbal behavior in human interactions and facial-affective behavior constitutes one of the primary channels for emotional communication. Facial-affective behavior constitutes an observable component of emotional processes (Bänninger-Huber and Widmer, 1999). Thus, it serves several functions for the regulation of the relationship, such as conveying information of the participant’s internal emotional states, and in that sense, they are a window for accessing unconscious and spontaneous affective states (Merten, 2005). They also communicate expectations about the interactive partner and about the relationship (including expectations regarding affect regulation), they serve the function of assessing and regulating the state of the relationship, of communicating emotional involvement, indicating how the individual copes with negative affect, and of attenuating, amplifying, simulating, emphasizing and enriching both verbal and non-verbal content of communication (Anstadt et al., 1997; Bänninger-Huber and Widmer, 1999; Dreher et al., 2001; Merten, 2005). Although a great portion of facial-affective cues occur too quickly for simultaneous verbal translation and conscious reflection to occur (Lyons-Ruth, 2000), the interactive partners are continuously reacting to the implicit as well as cognitive interpretations of these signals.

Although the study of facial affective behavior is transversal to several sub-disciplines of psychology, research on facial-affective behavior in psychotherapy suggests that relevant instances of the therapeutic exchange trigger varying degrees of emotional dysregulation in the therapy participants, with their concomitant attempts to self- and mutual regulation of these affective states. These attempts may be expressed both verbally and non-verbally (Bänninger-Huber, 1992; Bänninger-Huber and Widmer, 1999; Benecke and Krause, 2005). Specifically, non-verbal facial affective behaviors would involve a specific desire of regulation, as well as communicate certain attitudes toward the interactive partner or toward the state of the relationship, which include expectations about the interaction, and the interactive partner (Anstadt et al., 1997; Merten, 1997; Benecke and Krause, 2005; Rasting and Beutel, 2005). Inasmuch as the facial affective behavior or therapist and patient allows for the observation of affective regulatory processes that take place in the moment-by-moment exchange, this line of study stresses the interaction as a study unit.

The implementation of this research line initially involved the systematic study of cumulative single cases of psychotherapeutic processes of different theoretical orientations (see Barros et al., 2016; Altimir and Valdés-Sánchez, 2020); and more recently has broadened to a greater sample of therapies. It is relevant to note that although this research line is informed by relational psychoanalytic thinking, and it is particularly interested in accessing the implicit and unconscious portion of dyadic affective processes, so far it has been implemented on therapies of different theoretical orientations, including predominantly psychodynamic therapies. The rationale for this has been the consideration that both ruptures (Eubanks et al., 2018; Muran et al., 2021) as well as facial-affective behavior are a generic phenomenon that is present in all kinds of therapies. Although this may be considered a limitation of these studies, since these therapies are not based on manualized procedures, but instead are selected from naturalistic settings, it seeks to grasp what therapists and patients “actually do in ordinary therapies.” This is based on the fact that this research line is in the stage of opening a field of inquiry by progressively accumulating data from the “bottom-up,” placing a particular focus on the notion of affect regulation and implicit interactions. We expect these findings to generate new research questions and hypothesis about affect regulation that can further be examined within psychodynamic therapies, under the idea of systematically observing what psychoanalytic therapists and their patients do.

Psychotherapeutic dyads have been recruited through mental health care services and university based or psychotherapy training contexts, implying that most therapies have been brief and time-limited. All participants have been informed of the aims of the studies and have signed informed consents allowing the entire therapy to be videotaped, involving the installation of cameras in the room directed simultaneously toward therapist and patient. All videotaped therapy sessions have been observed by trained raters who code the presence of rupture and resolution strategy markers based on the Rupture Resolution Rating System (3RS) (Eubanks et al., 2015). Trained coders observed each therapy session and identified markers of both ruptures and resolution strategies according to the definition of the manual. This process yields a sample of ruptures and resolution strategy events along the total number of sessions of each therapy studied. These events last between two to several speaking turns (between seconds and 5 min long). It is important to note that not all therapy sessions contain rupture or resolution strategies, so the distribution of these events along the therapy process is not homogeneous. Thus, some sessions may contain two or three ruptures and one or no resolution strategy (which may address one of the ruptures or the sequence of ruptures in that session or even in previous sessions), while other sessions seem to focus on therapeutic work and no ruptures o resolution attempts are observed. Throughout this process, inter-rater reliability is estimated to assure adequate levels of observational validity.

From a process research perspective, these events constitute windows that allow an observational access into relevant interactions within psychotherapy, based on the significant events paradigm. This approach proposes to examine the specific moments of therapy that are considered relevant for change, as well as their components and the mechanisms that facilitate their occurrence (Greenberg, 1986; Safran, 2003). Thus, the psychotherapeutic process would be understood as a sequence of recurrent states and transitions between them, revealed in identifiable patterns of action. These events thus constitute “thick” experiential instances that provide significant information about the processes and mechanisms that form the foundations of psychotherapy (Safran, 2003). In a second moment, patient and therapist’s facial affective behavior within the previously identified rupture and resolution strategy events is coded by judges trained in the Facial Action Coding System (FACS) (Ekman and Friesen, 1978), an observational system that allows the objective coding of facial behavior. This behavior includes facial movements associated to the presence of basic emotions (happiness, anger, contempt, disgust, fear, sadness, and surprise), as well as behavioral indicators of emotional arousal and attempts at regulation (Anstadt et al., 1997), such as self-touching, control/attenuation of facial expressions, and elevation of eyebrows (Ekman, 1979). Since facial movements occur very rapidly, they were coded at a micro-level (Bänninger-Huber, 1992), implying that rupture and resolution strategy events were divided into 1-s-long video-frames (with.04 s resolution). This means that a rupture event 1 min long is divided into 60 frames. The presence of any facial-affective behavior within these frames is coded. In other words, if a micro-expression lasts 1 s, it is coded within the frame it takes place, if it lasts 45 s, it is coded in each of the 45 frames it takes place. To assure reliability on the FACs coding, inter-rater agreement was also estimated. This method is appropriate to capture facial-affective cues that occur at the split-second, and therefore are too fast to and automatic to undergo conscious awareness. Among these behavior, micro-expressions (Ekman, 2007) are fast facial movements lasting less than a quarter of a second and are involuntary. They are assumed to be the result of an intentional attempt at suppressing the true emotional experience or of unconscious repression, and therefore express an emotion the subject is totally unaware of. The relational context and the content of the interaction serve to clarify the meaning of both micro as well as macro-expressions (normal facial-affective expressions). In this case, the relational context of a rupture (tension in the relationship) or a resolution attempt (metacommunication and attempt at connecting), as well as the verbal content of these specific exchanges, will help attribute meaning to this behavior, and in that sense, its affective regulatory function.

The association between the participants’ facial-affective behavior within rupture and resolution strategy events were modeled by means of nested hierarchical regressions using Hierarchical Linear Modeling (HLM). A two-level model was estimated, where participants’ facial behavior was defined as the dependent variable (Level 1) and type of event as the predictor variable (Level 2). Also three-level models were estimated were facial-affective behavior was defined as the dependent variable and verbal relational offers as predictor variables (Level 2), while rupture/resolutions at Level 3 predictor variables. Separate nested models were estimated for the probability of occurrence of each Level-1 dependent variable. This method responds to the nested nature of the data, which HLM controls for. That is, each participant’s facial-affective behavior occurs within rupture/resolution events that in turn are nested within a same therapy and relationship, so they cannot be treated as independent variables. This quantitative approach allows for the analysis of a big number of events and frames, that can provide certain generalized conclusions about certain identified patterns. It is our aim to subsequently examine examples of these patterns in a more qualitative, process-descriptive manner.

Findings until now support the assumption that by studying facial-affective behavior, we can access the process of affect regulation involved in the intersubjective negotiation reflected in ruptures of the alliance and resolution attempts. Results of a sample of five psychotherapies, indicate that during withdrawal ruptures, patients displayed significantly more expression of positive emotions, mainly through the expression of social smile (Barros et al., 2016). In contrast to the expression of joy, which indicates felt happiness, social smile is not felt, but serves the function of maintaining a basic sense of security within the relationship and ensures a state of emotional resonance with the interactive partner (Bänninger-Huber, 1992; Benecke et al., 2005). It seems patients may have favored attempts to secure the bond that may have felt temporarily threatened by the rupture, prioritizing their needs for relatedness over their need for self-definition. Meanwhile, confrontation ruptures were characterized by an absence of + patients’ facial emotional correlate for the emotional experience of a confrontation rupture, perhaps indicating some kind of suppression, favoring their sense of agency over their need for relatedness.

A second single-case study which examined participants’ facial-affective communication in association to their verbal relational offers during ruptures and resolution strategies (Altimir and Valdés-Sánchez, 2020), indicates that the patient displayed significantly more negative emotions during ruptures, predominantly anger. Meanwhile, fear and indicators of regulatory behaviors attempting to control facial expression were more predominant during ruptures where the therapist’s relational offer was that of proposing a new perspective. At the same time, the patient avoided gazing at the therapist during ruptures, while verbally offering a receptive stance, and while therapist verbally offered a questioning and conciliatory stance, the patient displayed self-soothing behavior indicating emotional deregulation. These behaviors may indicate patient’s attempts to regulate emotional distance with the therapist. Meanwhile, patient’s facial-affective behavior during resolution strategy events indicates a consistent likelihood of patient gazing at him and of displaying markers of emotional deregulation. The therapist exhibited a sustained gaze at the patient, as well as emotional deregulation and attempts at controlling facial expression during rupture events. During resolution strategy events instead, therapist was more likely to display indicators of either making emphasis on his verbal/non-verbal communication or showing an interrogative stance.

Finally, a study by Altimir et al. (2017) based on a sample of five therapies of different theoretical approaches, indicate that ruptures are characterized by affect deregulation, emotional arousal markers and negative emotions in both patients and therapists, which in turn activate self-regulatory behaviors. Nevertheless, during these events, therapists sustain contact and emotional involvement with the patient through gazing at the interactive partner, while patients avoid contact through gaze. However, patients show more gazing toward their therapist during withdrawal ruptures compared to confrontation ruptures (Altimir et al., 2017). As facial affective research has specified, gaze behavior is a primary affective regulatory behavior in human emotional communication (Anstadt et al., 1997; Bänninger-Huber and Widmer, 1999; Dreher et al., 2001). Therefore, these findings indicate that gaze has an important function in regulating contact within relational tense events. Specifically, it seems that patient affect deregulation involves withdrawing from emotional involvement with the therapist during instances in which negotiation of the relationship triggers the unfulfillment or frustration of specific relational needs. On the other hand, the fact that therapists tend to sustain their gaze and therefore their involvement in the interaction, may indicate their ability to, at some extent, regulate their own emotional arousal and stay connected during the relational exchange, in spite that he/she may show other signs of emotional deregulation. This is coherent with the therapeutic model for addressing ruptures in the alliance developed by Safran and Muran (2000) and Muran et al. (2010), which suggests the relevance of the therapist affect regulation to allow the exploration of these events. Another relevant finding is that patients display more positive emotions, manifested in happiness/joy or through social smiles, during withdrawal ruptures compared to confrontation ruptures, specifically during withdrawal ruptures characterized by a content-affect split. This means the patient withdraws from the therapist and/or the work of therapy by exhibiting affect that does not match the content of his/her narrative (Altimir et al., 2017).

Although these results are based in a small number of cases, they indicate that it is possible to access observable indicators of participants’ attempts to regulate affective disturbance and deregulation within the therapeutic dyad. At the same time, alliance ruptures and resolution strategies constitute windows that provide a relational context to these self and mutual affective regulation processes, that allow making sense of these automatic and implicit behaviors. We have evidenced that ruptures trigger affective dysregulation, negative emotions and self-regulatory behaviors in both members of the therapeutic dyad and require therapist self-regulation to sustain relational involvement. In turn, resolution strategies are laborious processes of recognizing the difficult emotions triggered by the ruptures, involving an affective reflection of the patient’s internal experience and the therapist openness to explore patient’s underlying difficult affects. The future directions of this line of research are to confirm these findings examining a larger number of therapies, to progressively derive affective interactive patterns that characterize alliance ruptures and resolutions. At the same time, the aim is to achieve a greater specificity in the description of the affective regulatory processes across the different types of ruptures (withdrawal and confrontation), as they involve different relational movement in their attempt to negotiate the needs for relatedness and self-definition. Ongoing research in this line is currently attempting that endeavor.

## Clinical Implications of Research on Affect Regulation in the Analytic Practice

The clinical contribution of this line of research is that it provides systematic and empirical support to the relevance not only of the analytic relationship, but also of the clinical work that is focused on that relationship. Given that ruptures constitute a prevalent phenomenon within the therapeutic relationship (Eubanks et al., 2018; Muran et al., 2021), challenging its stability and the progress of therapy (Coutinho et al., 2011, 2014), it is relevant that therapists develop abilities to detect and address them in a timely manner. The model developed by Safran and Muran (2000), Muran et al. (2010) to identify and address them therapeutically already constitutes an important contribution for clinicians, by describing and systematizing a relational experience that is relevant to the analytic process. The fact that clinicians can count with specific tools to identify stressful relational events such as ruptures, favors an open attitude focused on the here and now of the relationship that allows working in an immediate and contingent manner. It also favors clinicians who privilege the relational approach to have concrete elements of their own and the patient’s internal states that allow focusing on the emergence of the immediate relational transactions that attempt to deal with those states, both at an explicit as well as implicit level.

Specifically in relation to the contribution of investigating affect self- and mutual regulation within rupture and resolution processes, we consider that the accumulation of systematic knowledge in this regard may imply a direct contribution to inform clinical practice as well as training. By incorporating a specific perspective on affect regulation to the description and understanding of the rupture-resolution process by means of facial-affective behavior highlights the relevant role of the affective processes in the analytic interaction. Clinical supervision and training can be informed by detailed observations of these interactions, for example, through videotaped therapies. The possibility of observing how verbal and facial-affective non-verbal dimensions of the patient-therapist intersubjective negotiation within ruptures and resolutions may help clinicians grasp the intertwined aspects of these dimensions. At the same time, it can help “train the clinical eye” to specific facial-affective and non-verbal cues that accompany these difficult relational events, to improve their identification and a timely approach to working on them.

The recognition that ruptures and resolution processes involve the activation of certain affective responses in both participants, indicates the importance of the therapist’s self-regulation process in tolerating and sustaining challenging relational events while being able to be open to what emerges from the emotional reactions of both participants and the possibility of regulating that experience. To the extent that the therapist is in charge of self-regulating his or her negative affective states and the disturbance aroused by the rupture, he or she is able to sustain the bond, in a connected way, and explore the course of the patient’s underlying experiences. This self-regulation is expressed in an accepting, flexible and responsive attitude toward the emergent aspects of the interaction, the new information and possibilities the ongoing interaction entails for the dyad (Safran and Muran, 2000). In turn, through facial-affective responses as well as verbal content, the therapist can offer the patient an alternative and more adaptive way of establishing close relationships (Benecke et al., 2005).

In this way, this capacity of the therapist acts as an interactive regulation of the patient’s dysregulation, opening up new areas of inquiry and new ways of relating to the patient. The therapist’s capacity to self-regulate his own internal affective states in order to explore the patient’s mind is the basis of any mentalizing process. The exploration of the affects triggered by the rupture allows the dyad to mentalize the experience of the patient and of the interaction, thus, the negotiation of each other’s needs for relatedness and self-definition.

At the same time, the possibility of counting with facial indicators that characterize rupture events and resolution attempts, and eventually being able to distinguish particular and distinctive indicators for the different types of rupture, can help the clinician to be more attentive to the patient’s dysregulation processes as well as to orient his/her exploration toward more specific contents associate to the experience of disturbance. This responds to the assumption that different withdrawal and confrontation ruptures express particular ways of dealing with the disturbance in the intersubjective negotiation process, and therefore, may be expressed differently though facial-affective behavior. In that sense, this line of research may contribute to better describe ruptures that may often go unnoticed by patient and therapist (Safran et al., 2011), such as withdrawals, and help clinicians be more attentive to specific markers of such processes. In similar vein, the possibility of describing the therapist’s emotional reactions can contribute to foster greater self-awareness and attention to the therapist’s own reactivity an internal state, and therefore to countertransferential processes. This is in line with a mindful attitude toward the present experience, particularly to the which is embodied, by incorporating one’s own facial expression as a source of information on these internal states. This in turn favors therapist self-regulation and therefore the process of relational understanding of the emerging interaction. Having observable facial markers in the patient, as well as self-perceived in the therapist’s own facial expression can thus favor clinicians to be attentive to the oscillations experienced in the therapeutic relationship, and to address them more effectively. This is relevant inasmuch as ruptures require an adequate therapeutic approach and the possibility of addressing them for their positive resolution, which is fundamental for the continuity of the therapy and a fruitful therapeutic work (Coutinho et al., 2011, 2014).

## Discussion

Psychoanalysis marked the birth of psychotherapy as a discipline. However, the history of its relationship with academia and to the scientific method of acquiring knowledge remains controversial. In recent decades, however, a growing consensus has emerged that psychoanalysis is a discipline that should draw on both hermeneutic *and* scientific disciplines (Strenger, 1991). After decades in which the focus of clinical research was on the unconscious manifestations of the patient in the transference to the analyst, attention has turned to the intersubjective relationship between patient and therapist. Psychoanalysis has thus undergone a relational and phenomenological turn, where what is relevant is the immediacy of the analyst-patient interaction. Traditional clinical studies do not do justice to the complexity of the analytic relationship, as they are based on verbal reports reconstructed from memory and therefore cannot capture the immediacy of the relationship. In doing so, they leave out the possibility of grasping the moment-to-moment unfolding of the interpersonal exchange, and therefore to access what “psychoanalysts actually do.” This also includes the possibility of accessing the non-verbal, implicit, and procedural realm of the intersubjective process. Thus, the Freudian metaphor of telephone communication as a representation of unconscious-to-unconscious communication is left out of clinical and scientific exploration. Yet, there is no distinct or direct method to study unconscious and implicit phenomena, since it is always subject to interpretation. Our proposal to study participants’ facial-affective behavior is assumed to be an innovative method to access affective regulation of the analytic interaction, as it attempts to relate unconscious phenomena with symbolic and complex meaning making, that is, the “in-between” processes. We believe that by simultaneously examining verbal intentional affective states and linking them with non-verbal affective cues, we may contribute to grasp and perhaps do justice to the central idea of psychoanalytic process, that is, the relevance of the unconscious in therapeutic work.

Therefore, an innovative research program should address the empirical study of the processes that take place between patient and therapist, paying special attention to the manifestations of the implicit domain of the relational experience, as it can shed light into unconscious phenomena. In this paper we have described that one of the most relevant findings of contemporary neuroscience are the implicit phenomena, i.e., those that are out of consciousness, and account for more than 90% of mental life. Implicit phenomena play a relevant role in the patient–therapist affective communication. Most relational exchanges are strongly based on affective cues that contain specific information about emotional states and cognitive appraisal processes, which are captured and utilized by both participants at the instant. Within this communication, affective signals take place in fragments of seconds, so that the speed and density of the information that is being exchanged does not allow the central control of cognition, that is, a verbal translation and conscious reflection. An innovative research program must be able to capture the implicit level, the moment-by-moment exchange, the interactive emotional patterns of facial behavior, gaze, vocalization, and orientation of the participant simultaneously.

Our research strategy chooses the simultaneous capture and analysis of the verbal contents and the interaction of gestures and glances, as an expression of the implicit unconscious exchange. Of course, it is possible to add other expressions, such as voice and body gestures. The progress of research in this field, as well as the systematic study of long therapies and in particular clinical populations (e.g., personality disorders) will enrich the knowledge of clinical exchange. We believe that only in this way the accumulation of clinical knowledge, in permanent dialogue with scientific findings, will lead psychoanalysis to a unified scientific discipline, as conceived by Freud in his 1923 article.

## Data Availability Statement

The original contributions presented in the study are included in the article/supplementary material, further inquiries can be directed to the corresponding author/s.

## Ethics Statement

The data referred to in the manuscript are secondary data from previous studies duly authorized by ethics committees. The patients/participants provided their written informed consent to participate in this study.

## Author Contributions

All authors listed have made a substantial, direct, and intellectual contribution to the work and approved it for publication.

## Conflict of Interest

The authors declare that the research was conducted in the absence of any commercial or financial relationships that could be construed as a potential conflict of interest.

## Publisher’s Note

All claims expressed in this article are solely those of the authors and do not necessarily represent those of their affiliated organizations, or those of the publisher, the editors and the reviewers. Any product that may be evaluated in this article, or claim that may be made by its manufacturer, is not guaranteed or endorsed by the publisher.
